# Quantitative CT-based image registration metrics provide different ventilation and lung motion patterns in prone and supine positions in healthy subjects

**DOI:** 10.1186/s12931-020-01519-5

**Published:** 2020-10-02

**Authors:** Kyung Min Shin, Jiwoong Choi, Kum Ju Chae, Gong Yong Jin, Ali Eskandari, Eric A. Hoffman, Chase Hall, Mario Castro, Chang Hyun Lee

**Affiliations:** 1 Department of Radiology, School of Medicine, Kyungpook National University, Kyungpook National University Chilgok Hospital, Daegu, South Korea; 2 Department of Internal Medicine, School of Medicine, The University of Kansas, Kansas City, Kansas, USA; 3 Department of Bioengineering, The University of Kansas, Lawrence, Kansas, USA; 4 Department of Radiology, Research Institute of Clinical Medicine of Jeonbuk National University-Biomedical Research Institute of Jeonbuk National University Hospital, Jeonju, South Korea; 5 Department of Radiology, University of Iowa, Iowa City, USA; 6 Internal Medicine, University of Iowa, Iowa City, USA; 7 Biomedical Engineering, University of Iowa, Iowa City, USA; 8 Department of Radiology, Seoul National University College of Medicine, Institute of Radiation Medicine, Seoul National University Hospital, 101 Daehak-ro, Jongnogu, Seoul, 03080 South Korea

**Keywords:** Acute respiratory distress syndrome (ARDS), Prone positioning, Quantitative computed tomography (QCT), Lung motionography

## Abstract

**Background:**

Previous studies suggested that the prone position (PP) improves oxygenation and reduces mortality among patients with acute respiratory distress syndrome (ARDS). However, the mechanism of this clinical benefit of PP is not completely understood. The aim of the present study was to quantitatively compare regional characteristics of lung functions in the PP with those in the supine position (SP) using inspiratory and expiratory computed tomography (CT) scans.

**Methods:**

Ninety subjects with normal pulmonary function and inspiration and expiration CT images were included in the study. Thirty-four subjects were scanned in PP, and 56 subjects were scanned in SP. Non-rigid image registration-based inspiratory-expiratory image matching assessment was used for regional lung function analysis. Tissue fractions (TF) were computed based on the CT density and compared on a lobar basis. Three registration-derived functional variables, relative regional air volume change (RRAVC), volumetric expansion ratio (*J*), and three-dimensional relative regional displacement (s*) were used to evaluate regional ventilation and deformation characteristics.

**Results:**

*J* was greater in PP than in SP in the right middle lobe (*P* = 0 .025), and RRAVC was increased in the upper and right middle lobes (*P* < 0.001). The ratio of the TF on inspiratory and expiratory scans, *J*, and RRAVC at the upper lobes to those at the middle and lower lobes and that ratio at the upper and middle lobes to those at the lower lobes of were all near unity in PP, and significantly higher than those in SP (0.98–1.06 vs 0.61–0.94, *P* < 0.001).

**Conclusion:**

We visually and quantitatively observed that PP not only induced more uniform contributions of regional lung ventilation along the ventral-dorsal axis but also minimized the lobar differences of lung functions in comparison with SP. This may help in the clinician’s search for an understanding of the benefits of the application of PP to the patients with ARDS or other gravitationally dependent pathologic lung diseases.

**Trial registration:**

Retrospectively registered.

## Background

The concept of prone positioning of patients on mechanical ventilation for ARDS was proposed in the 1970s and has been regarded as a rescue strategy. Prone position (PP) has been shown to improve oxygenation [1–4] and survival [5–7] in patients with ARDS. Despite the clinical benefits of proning a patient, the mechanism of improvement is not completely understood. The differences in the supine position (SP) versus PP might be related to some anatomical and physiological parameters. From the gravity oriented SP gradients in lung expansion and the considerable reduction in this gradient, not just the reversal of the expansion gradient, in PP allows one to deduce that pleural pressure gradient from nondependent to dependent regions are less in the PP, in part by gravitational effects, reducing the superimposed pressure of both the heart and possibly the abdomen. In a comparative physiology study, the authors concluded that the support of the heart by the lungs in the SP versus support of the heart by the sternum in the PP, rather than shape changes in the rib cage and diaphragm, may be responsible for the differences in resting, recumbent lung expansion gradients [8]. More recent works in humans have confirmed the reversal and considerable reduction in resting gradient in ventral-dorsal lung expansion when shifting a human from the supine to the prone body posture [9, 10]. In contrast to effects on lung aeration, pulmonary blood flow is constantly directed dorsally in normal and injured lungs in both the SP and the PP [3, 11]. Therefore, the PP improves ventilation-perfusion matching [12], and contributes to improvement in oxygenation. During mechanical ventilation, the more uniform distribution of lung strain allows for recruitment at lower positive pressure, a reduction in regional shear strain, and thus less ventilator-induced lung injury (VALI) [13, 14].

On the basis of these observations, the analysis of regional heterogeneity of aeration and strain, taking advantage of advances in CT technologies, is crucial for understanding the mechanism of the clinical benefit of the PP. Advances in quantitative computed tomography (CT) imaging and data analysis techniques allow clinicians to assess regional functional and mechanical metrics such as ventilation and regional lung deformation in considerably greater detail [15–17], and these techniques provide maps of regional ventilation and lung stretch between pairs of inspiratory and expiratory images in three-dimension [18, 19]. To obtain objective results of CT quantitative analysis, a method of automated lung CT segmentation is needed. However, the segmentation of ARDS involvement requires time and experience because consolidation or atelectasis of the lung limits structural reference contrast against the chest wall [18, 20]. Furthermore, transport of critically ill patients to a CT scanner is problematic [21]. Although density-based CT quantification of the SP versus PP has been explored in experimental [22] and human studies [13, 23–25], functional and mechanical CT metrics have not been used to compare the differences between the SP and the PP.

Therefore, we hypothesized that through the use of three-dimensional (3D) CT image registration in healthy subjects, quantification of regional ventilation metrics and visualization of the regional lung motion could clarify the benefit of the PP demonstrated in ARDS. The purpose of this study was to quantitatively compare the regional characteristics of lung functions on a lobar basis between PP and SP with the use of paired inspiratory and expiratory CT scans.

## Methods

Our institutional review board approved this retrospective study and waived the requirement for informed consent.

### Patients

We recruited patients with normal pulmonary function test who had undergone chest CT scanning from the data set that were originally collected for quantitative analysis of lungs in normal subjects [26]. We used the following inclusion criteria: (1) availability of both inspiratory and expiratory CT scans; (2) no known history of lung disease or surgery; and (3) absence of nodules or less than 5 nodules (< 4 mm). Fifty-six subjects (26 men, 30 women; mean age, 50.5 ± 14.7 years) were included retrospectively for the SP. In addition, we retrospectively collected 34 subjects (16 men, 18 women; mean age, 47.4 ± 10.6 years) for the PP who performed follow up CT in PP to differentiate subpleural nodules or interstitial abnormality from gravity-dependent atelectasis or nodular atelectasis in the dependent regions of the lungs for the clinical purposes.

### CT acquisition

All patients underwent 128 multidetector CT scanning (Somatom Definition Flash; Siemens Healthcare, Forchheim, Germany) under full inspiration and full expiration. CT parameters were as follows: tube voltage (120 kVp), tube current (inspiration, 110 mAs; expiration, 50mAs), slice thickness (1.0 mm), and reconstruction interval (1.0 mm), reconstruction algorithm (B35f), rotation time (0.5 s). Inspiratory scans (IN) were obtained at total lung capacity (TLC), and expiration scans (EX), at functional residual capacity (FRC).

### Image registration, regional air volume change, and lung deformation

**Fig. 1:**
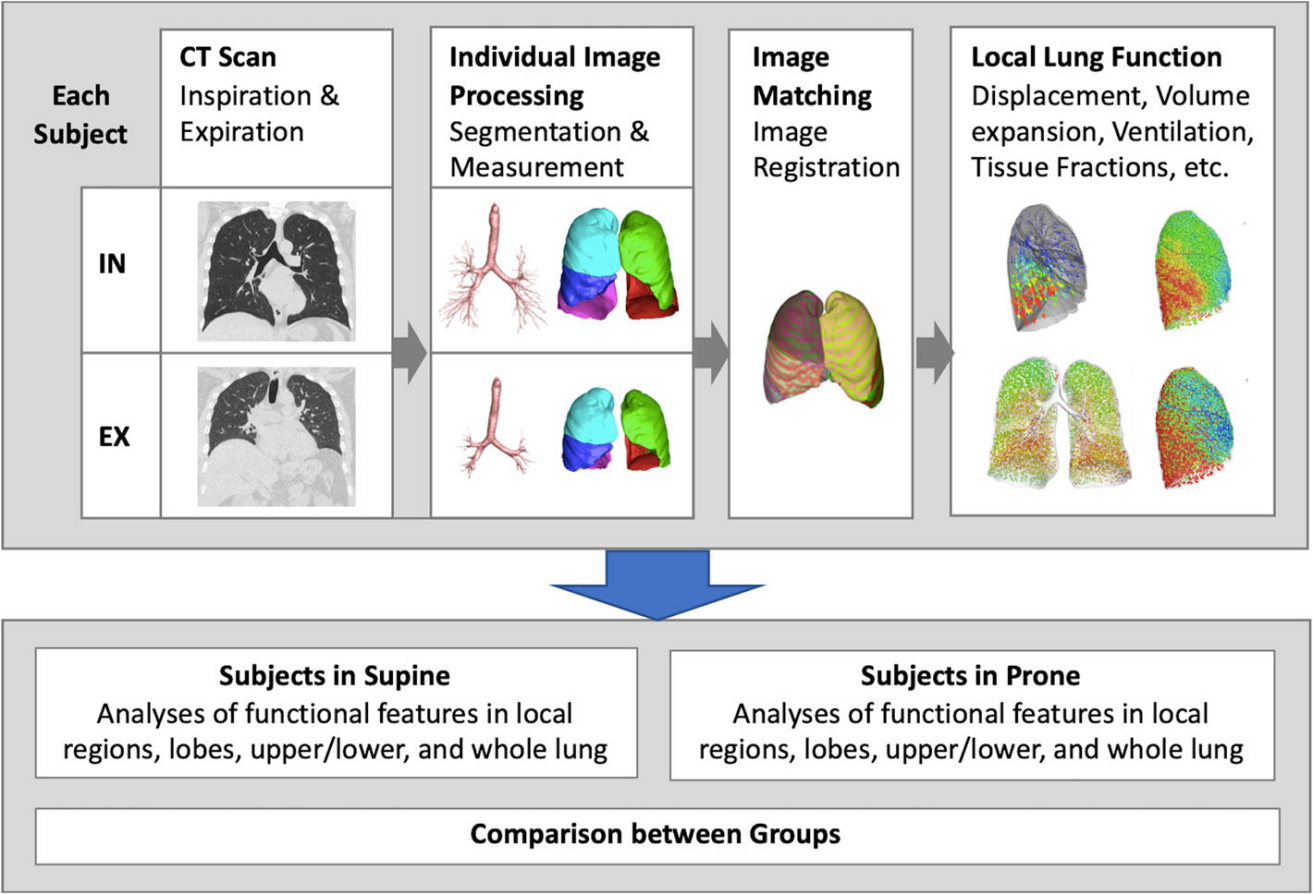
Flow chart of the steps involved from image acquisition to the regional lung functional maps, and further to the comparison between supine and prone groups

**Fig. 2:**
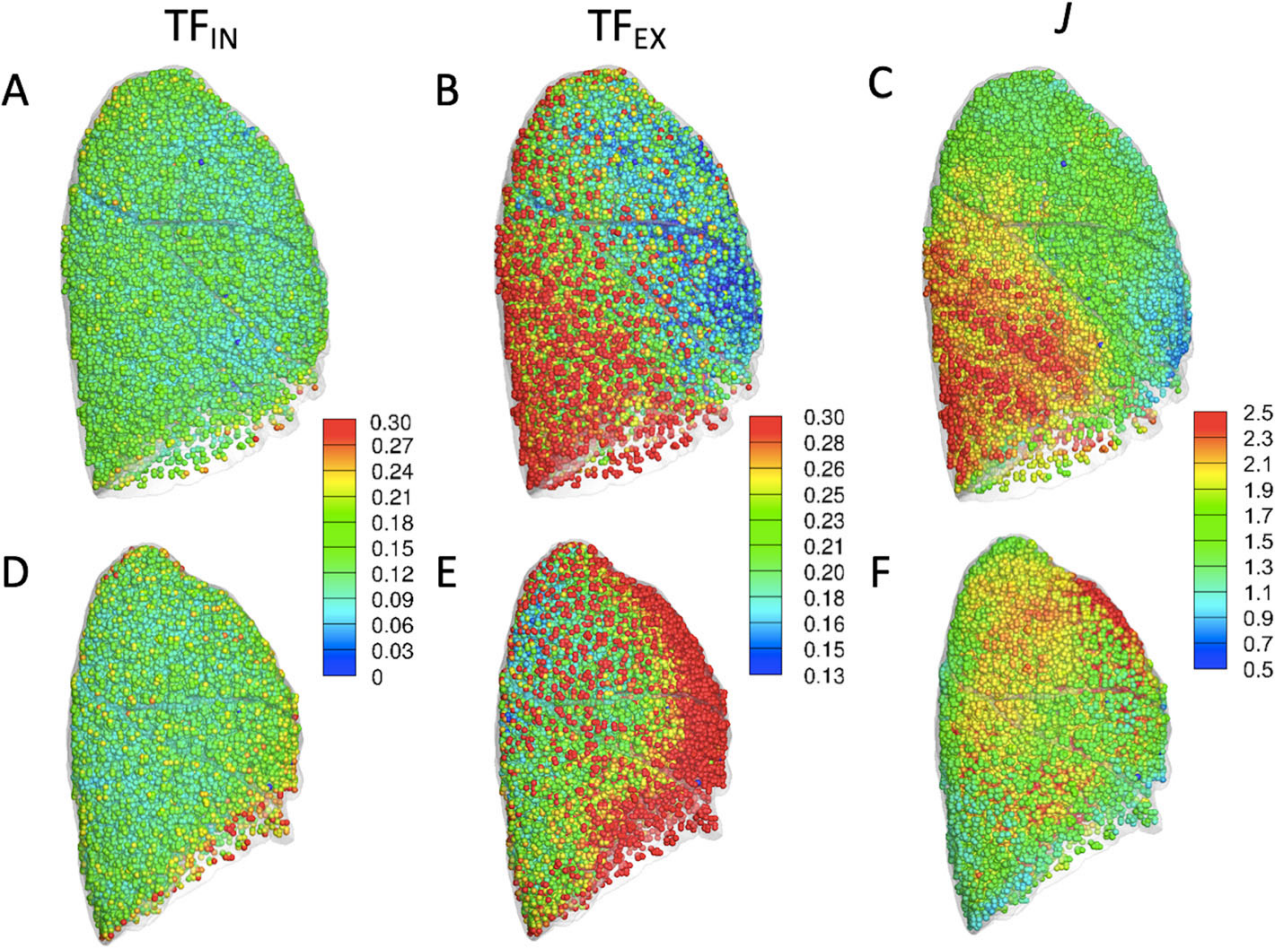
Demonstration of inspiratory (**a** and **d**) and expiratory (**b** and **e**) tissue fraction (TF_IN_ and TF_EX_, respectively) maps and local expansion (*J*) map (**c** and **f**) in supine (**a-c**) and prone (**d-f**) positions*.* TF_IN_*,* TF_EX_, and *J* were computed in local lung parenchymal volumetric units at an acinar scale and were colored according to a rainbow scale. TF_IN_ and TF_EX_ are plotted in the same scale for comparison between inspiration and expiration. Since the summation of both air and tissue fractions is equal to unity, the decrease in tissue fraction implies an increase in air fraction. *J* is computed as inspiratory-to-expiratory volume ratio at matched parenchymal units

Figure 1 is the flow chart of the steps involved from image acquisition to the regional lung functional maps. All volumetric CT images at inspiration and expiration were segmented with the use of VIDA Apollo pulmonary workstation, version 2.0 (VIDA Diagnostics, Coralville, IA, USA). The mass-preserving nonrigid image registration technique was employed to match local lung regions (voxels) of inspiratory and expiratory CT images [15]. Local air and tissue volumes as well as tissue fraction (TF) were computed in voxels based on the CT density and compared on a lobar basis [16]. Image registration provided local displacement vectors between the matched points in the paired two images and displacement magnitude was normalized by the cubic root of lung volume change between two images to reduce the influence of different inflation levels among subjects (Fig. 2). Normalized 3D relative regional displacement was denoted by s*. From matched units of local lung parenchyma on an acinar scale, 3D displacement vectors from expiration to inspiration were computed. Local volume change ratio of inspiratory to expiratory images (computed by Jacobian determinant, *J*) and relative regional air volume change (RRAVC) were derived to quantify lung deformation [27, 28]. Thus, s*, *J*, and RRAVC were indicators of preferential lobar nonuniform stretch (a measure of the magnitude of direction preference in volume change), local expansion (a measure of regional volume change), and air-volume change (a measure of regional ventilation), respectively [16]. Voxel-scale local lung variables were presented in approximately 30,000 acinar-scale parenchymal units to exhibit lung function distribution. The ratio of the CT variables at the upper lobes to those at the middle and lower lobes (U/ML) and the ratio of those at the upper and middle lobes to those at the lower lobes (UM/L) were calculated in PP and SP, respectively.

### Statistical analysis

Data were expressed as means (± standard deviations). Student’s *t*-tests were used to compare the functional metrics of inspiratory and expiratory CT scans between the SP and the PP. A *P* value of less than 0.05 was considered significant. Software R (R Foundation for Statistical Computing, Vienna, Austria) was used for statistical analyses.

## Results

### Tissue fractions in supine vs prone

Table 1 summarizes the results of the changes in TFs according to the lobes in the SP and PP. TF on inspiratory scans (TF_IN_) was decreased in the lower lobes (*P* < 0.003) and increased in the right middle lobe in the PP (*P* < 0.001). TF on expiratory scans (TF_EX_) was greater in the right middle lobes and the whole lung (*P* < 0.01) but not in the lower lobes (*P* > 0.05) in the PP. UM/L and U/ML of TF_IN_ and TF_EX_ were all near unity in PP, whereas they are smaller in the SP (*P* < 1.0 × 10^− 7^; Table 2). In both SP and PP, the inspiratory image shows a relatively uniform distribution of TF that is attributable to the full recruitment of alveoli (Fig. 2a and d). On expiration, the gradient toward gravitationally dependent dorsal and basal regions in SP was shifted toward ventral regions (Fig. 2b and e).

**Table 1:** Results of Tissue Fractions in Prone Versus Supine Positions

Variable	Region	Prone	Supine	*P* value
TF_IN_	Upper	0.151	0.150	0.713
	RML	0.167	0.142	1.8 × 10^−3^
	Lower	0.147	0.163	0.003
TF_EX_	Upper	0.253	0.235	0.127
	RML	0.277	0.211	1.5 × 10^−6^
	Lower	0.243	0.295	0.001

All values are expressed as means. *RML* Right middle lobe, *TF*_*IN*_ Tissue fraction on inspiratory scan, *TF*_*EX*_ Tissue fraction on expiratory scan

**Table 2:** Upper to Lower Lobes Ratio of TF_IN_, TF_EX_, and *J* in Prone Versus Supine Positions

Variable	UM/L			U/ML		
	Prone	Supine	*P* value	Prone	Supine	*P* value
TF_IN_	1.06	0.91	1.4 × 10^−12^	1.02	0.94	2.1 × 10^− 8^
TF_EX_	1.06	0.79	3.6 × 10^− 19^	1.02	0.84	3.7 × 10^−17^
*J*	1.00	0.86	2.4 × 10^−24^	1.01	0.89	2.1 × 10^−19^
RRAVC	0.99	0.61	6.6 × 10^−4^	0.98	0.78	4.8 × 10^−9^

All values are expressed as means. *J* Jacobian, *RRAVC* Relative regional air volume change, *TF*_*EX*_ Tissue fraction on expiratory scan, *TF*_*IN*_ Tissue fraction on inspiratory scan, *U/ML* Ratio of variables of upper lobe to those of middle and lower lobes, *UM/L* Ratio of variable of upper and middle lobes to that of lower lobe

### Regional lung volumetric expansion ratio (J) and motion (s*) in supine vs prone

**Fig. 3:**
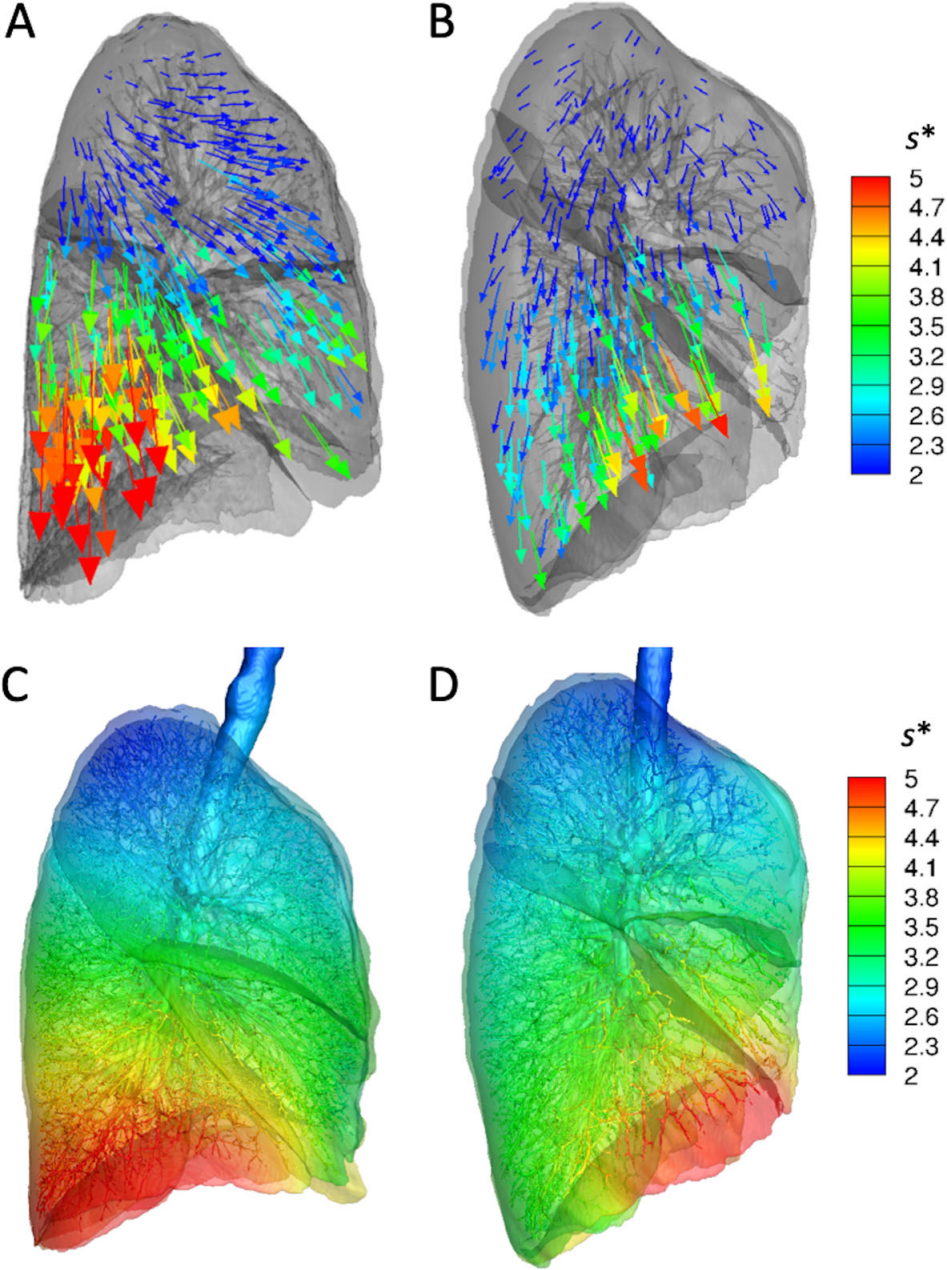
Demonstration of regional lung motionography in supine and prone positions. Displacement vectors from expiration to inspiration (**a** and **b**) and normalized 3D displacement magnitude maps on entire lung regions (**c** and **d**) in supine position (**a** and **c**) and prone position (**b** and **d**). For visual interpretation, three-dimensional displacement vectors were plotted and colored according to a rainbow scale by s*

**Fig. 4:**
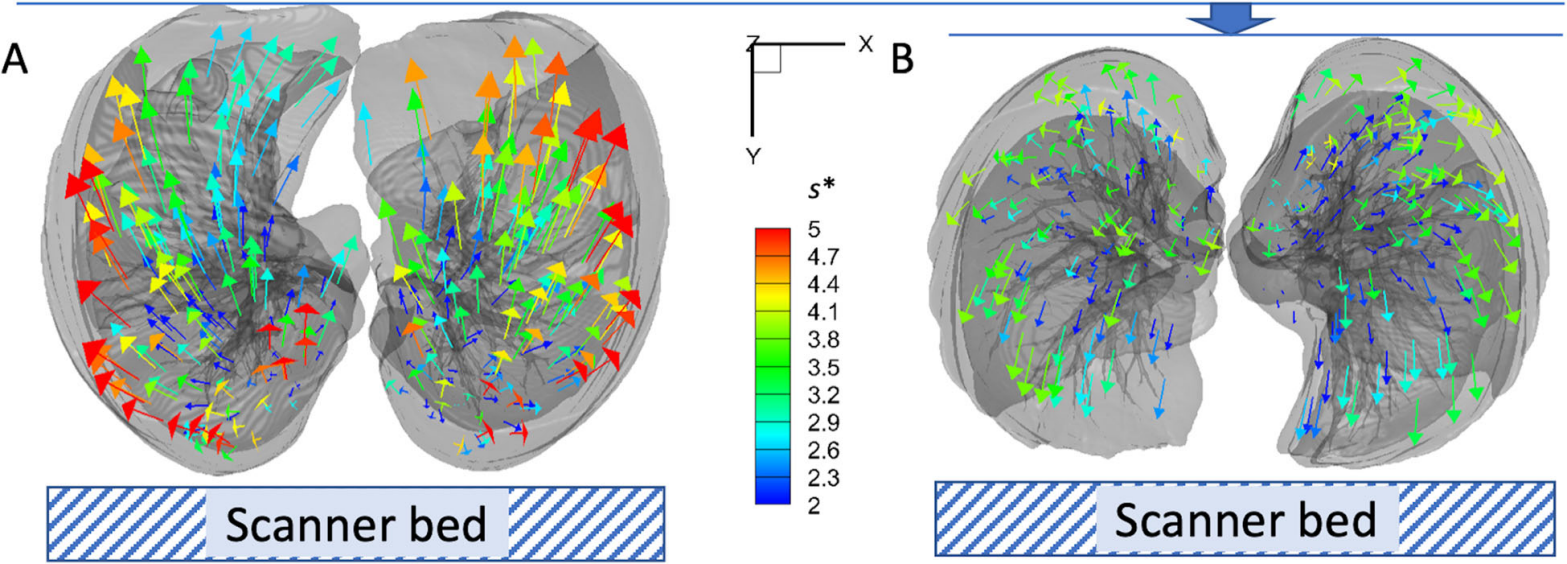
Bottom view comparison of displacement vector distributions in (**a**) supine and (**b**) prone positions of the same subject (M, 53 yr) for demonstration

In the PP, *J* was decreased in the lower lobes (*P* < 0.005) and increased in the right middle lobe (*P* = 0.025; Table 3), as demonstrated in Fig. 2f compared with Fig. 2c. In the SP, the regional displacement gradient increased toward dorsal-basal regions. However, it was reduced and shifted toward anterior regions in the PP (Fig. 3). From one subject who had both supine and prone CTs, motionography in an axial view from the bottom (diaphragm) was compared in Fig. 4. The eminent decrease of regional difference and a slight decrease of anterior-posterior distance were observed. U/ML ratios of s* were decreased in the PP (*P* = 0.004), but UM/L ratios were not. UM/L and U/ML ratios of *J* were near unity and significantly greater than those in SP (*P* < 1.0 × 10^− 18^; Table 2).

**Table 3:** Regional volume expansion ratio and air volume change in prone versus supine positions

Variable	Region	Prone	Supine	*P* value
*J*	LUL	1.613	1.551	0.301
	LLL	1.600	1.809	0.001
	RUL	1.598	1.552	0.448
	RML	1.567	1.447	0.025
	RLL	1.597	1.797	0.002
RRAVC	LUL	1.040	0.891	7.6 × 10^−7^
	LLL	1.069	1.270	4.8 × 10^− 7^
	RUL	1.012	0.890	6.4 × 10^−4^
	RML	1.049	0.713	4.4 × 10^−19^
	RLL	1.058	1.279	7.2 × 10^−4^

All values are expressed as means. *J* Jacobian, *LLL* Left lower lobe, *LUL* Left upper lobe, *RLL* Right lower lobe, *RML* Right middle lobe, *RRAVC* Relative regional air volume change, *RUL* Right upper lobe

### RRAVC in supine vs prone

**Fig. 5:**
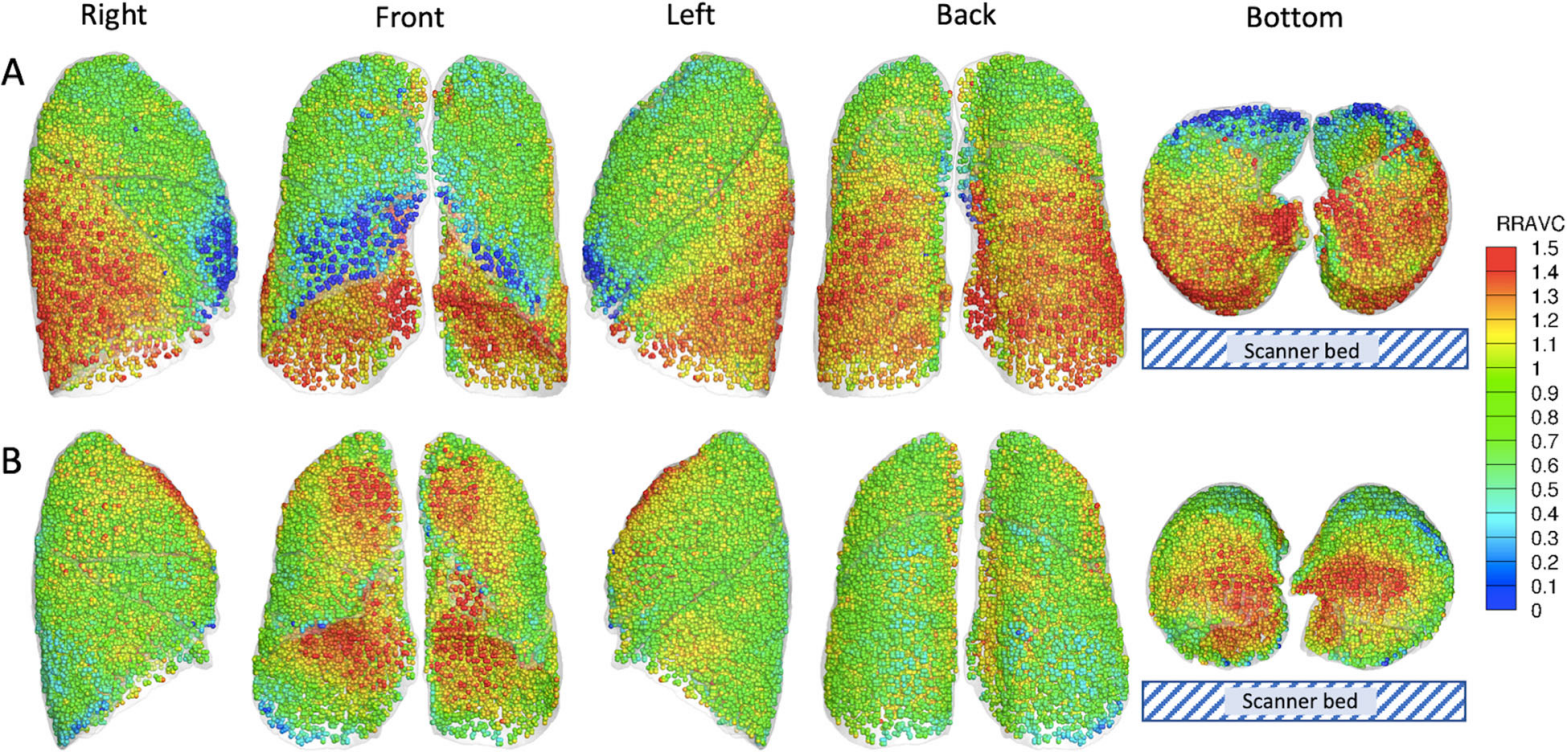
Demonstration of RRAVC between supine (**a**) and prone (**b**) positions from five directions (right, front, left, back, and bottom). RRAVC was computed in local lung parenchymal volumetric units at an acinar scale and was colored according to a rainbow scale. A region with an RRAVC value greater (or less) than 1 receives more (or less) ventilation than the mean over the whole lung. RRAVC, relative regional air volume changes

RRAVCs increased in the upper lobes (*P* = 6.7 × 10^− 6^) and decreased in the lower lobes (*P* = 3.3 × 10^− 5^) in the PP, in comparison with those in SP. Figure 5 shows RRAVC distributions in five different views in demonstrative subjects in supine and prone positions. In SP, a strong gradient toward gravitationally dependent regions is observed. On the contrary, the gradient is neutralized in PP, with a shift of ventilation contribution toward ventral regions. In the PP, UM/L and U/ML ratios of RRAVCs (0.99 and 0.98, respectively) were near unity and significantly higher than those in the SP (0.61 and 0.78, respectively; *P*s = 6.6 × 10^− 4^ for UM/L ratios and 4.8 × 10^− 9^ for U/ML ratios).

## Discussion

In this study, we performed a quantitative analysis to compare regional characteristics of lung functions between the SP and PP by using image registration applied to matched pairs of inspiratory and expiratory CT scans. We derived functional CT metrics by quantifying lung deformation and computed the regional tissue deformation and mechanics on a lobar basis. This study visualized that PP induced more uniform contributions of regional lung ventilation on RRAVC maps and a shift of 3D s* toward anterior regions on the 3D motionography. We calculated the ratio of functional measures in each lobe and found that the PP minimized the lobar differences in lung functions.

In several previous studies, researchers attempted to develop a hypothesis of homogeneous ventilation in the PP by using density-based CT analysis. Studies with standard CT volumetry showed that the vertical gradient CT density was attenuated when patients with ARDS were in PP [8, 24], and the cephalocaudal gradient was reduced in experimental animal models [22]. These results indicate that the PP induces a more uniform distribution of gas and tissue by reducing anteroposterior and cephalocaudal gradients. Other studies showed that the volume of overinflated lung mass decreased, whereas the volume of nonaerated or poorly aerated lung mass increased in the PP, which suggests that lung recruitment and homogeneity were enhanced in the PP [13, 23]. In this study, we computed tissue fractions (TF_IN_ and TF_EX_) rather than the CT density-based volumetry to quantify the regional homogeneity.

The present study demonstrates that, in the PP, TF_IN_ and TF_EX_ were increased in the right middle lobe but decreased in the lower lobes in the PP (*P* < 0.05; Table 1). In addition, UM/L ratios of TF_IN_ and TF_EX_ were all near unity in the PP, and were significantly greater than those in the SP (*P* < 1.0 × 10^− 7^). These findings corroborate the previous results from density-based CT volumetry studies with regard to homogeneity in the PP. This improvement in ventilatory homogeneity in ARDS leads to a decrease in the shunt fraction [3, 4], and in approximately 70% of patients, ventilation-perfusion mismatching and oxygenation were markedly improved in the PP [3, 4]. However, improvement in oxygenation in the PP does not fully explain the decreased mortality of ARDS [29]. Thus, the survival benefit observed with the PP is assumed to be more related to a protective effect against VALI [10, 30]. Indeed, recent data indicate that the paradigm of ARDS management has shifted from improving gas exchange to minimizing VALI and ensuring lung protection [31].

ARDS is characterized by a massive loss of lung aeration for tidal ventilation, which predominates in dorsal regions in the SP [32]. Hence, the mechanical distortion and regional overdistension caused by tidal ventilation develop in “ventral aerated lungs” in the SP, which is the most important determinant of VALI [33]. The PP has been reported to delay and attenuate the progression of VALI in animal studies [14, 30], and a CT volumetric study in patients with ARDS also revealed that PP was an effective lung protective strategy that could possibly alleviate VALI [13]. However, these studies did not provide evidence of CT metrics with regard to reduced strain in the PP. A more recent experimental study with inspiratory and expiratory CT scans revealed that the PP alleviated lung injury by minimizing the patterns with suboptimal aeration and large tidal swings (termed “unstable inflation”) [34]. The investigators employed parametric response maps, which have an advantage of spatial localization compared with simple density maps, and can depict regional heterogeneity. However, simple density difference measures are confounded by the effect of the baseline inflation of the target lung region. Therefore, we used the functional CT metrics of regional ventilation and tissue mechanics including s*, *J*, and RRAVC, which enable us to detect additional heterogeneity of lung strain and stress over CT density map [16, 35].

Our results showed that UM/L and U/ML ratios of RRAVC and *J* were all near unity in the PP and significantly higher in the PP than in the SP (*P*s < 0.001; Table 2). Moreover, the regional displacement gradient was reduced and shifted toward the anterior regions in the PP (Fig. 3). Because the loss of lung aeration is distributed mainly in the dorsal and caudal lungs in the SP [36, 37], these results together suggest that regional differences in strain between lung regions are reduced in the PP. Given that it is a crucial step to decrease lung strain in ARDS to prevent VALI, the PP could be an effective strategy of recruiting nonaerated lung and preventing overinflation. Nonetheless, the PP has been shown to be effective and is recommended in only severe ARDS [6, 38]. It is speculated that the PP benefits more hypoxemic patients with ARDS, who have more severe and heterogeneous lung injury and greater ventilation–perfusion mismatch. However, with increasing understanding of the heterogeneous pathophysiology of ARDS, identifying patients who will most benefit from the PP remains elusive [10]. Recently, lung morphology, assessed by CT images, has been suggested as a predictor of benefit from the PP, which indicates that gravity dependent ARDS is more likely to respond to the PP than is diffuse ARDS [13, 39]. Of note was that a lobar analysis in our study showed a trend for RRAVC and *J* to increase in the right middle and upper lobes and to decrease in the lower lobes in the PP (Table 3). Moreover, our study included the 3D motionography and mapping of the regional lung homogeneity and strain. In the real-life clinical practice for ARDS, lung morphology assessments are not readily available and frequently misclassified [31], and so the visual information provided in this study could serve as a surrogate to apply personalized PP therapy in patients with gravity dependent ARDS.

This study had some limitations that need to be considered. First, a comparison of CT measures between the SP and PP was performed mostly in different subjects. Thus, intersubject variability may have affected CT measures. However, we included only subjects with normal lungs. In addition, the distributions of specific volume change and density have been demonstrated to be more homogeneous in healthy subjects than in patients with lung disease [40]. Second, this quantitative analysis was in healthy subjects with normal lungs, not in patients with ARDS. In addition, inspiratory scans were taken at TLC, i.e., with a volume excursion several times larger than the tidal volume used during mechanical ventilation. Hence, the effect of CT metrics extracted from homogeneous normal lungs taken at TLC could be a little different in heterogeneous ARDS during mechanical ventilation. Indeed, the PP is known to be effective only in severe ARDS, and identifying patients who would most benefit from the PP remains a clinical challenge [38]. Therefore, further research into the comparison of these regional ventilation and lung mechanics between the SP and PP in ARDS is warranted.

## Conclusions

In conclusion, we visually and quantitatively observed that the PP not only induced more uniform contributions of regional lung ventilation along the ventral-dorsal axis but also minimized the lobar differences in lung functions compared with the SP. These findings along with the methodologies used in this study may help in the clinician’s search for an understanding of the benefits of the application of PP to the patients with ARDS or other gravitationally influenced pathologic lung diseases.

## Data Availability

The datasets used and/or analysed during the current study are available from the corresponding author on reasonable request.

## Abbreviations

ARDS: Acute respiratory distress syndrome
CT: Computed tomography
FRC: Functional residual capacity
*J*: Jacobian determinant
PP: Prone position
RRAVC: Relative regional air volume change
*s**: Three-dimensional relative regional displacement
SP: Supine position
TF_IN_: Tissue fraction on inspiratory scan
TF_EX_: Tissue fraction on expiratory scan
TLC: Total lung capacity
VALI: Ventilator-associated lung injury
